# Neurosensory mechanotransduction through acid-sensing ion channels

**DOI:** 10.1111/jcmm.12025

**Published:** 2013-03-14

**Authors:** Chih-Cheng Chen, Chia-Wen Wong

**Affiliations:** a Institute of Biomedical Sciences, Academia SinicaTaipei, Taiwan; b Institute of Zoology, National Taiwan UniversityTaipei, Taiwan; c Taiwan Mouse Clinics-National Phenotyping and Drug Testing Center, National Research Program for Biopharmaceuticals, National Science CouncilTaipei, Taiwan

**Keywords:** ASIC, MEC, DRG, mechanotransduction, mechanoreceptor, touch, pain, mechano-clamp, mechanically activated current

## Abstract

Acid-sensing ion channels (ASICs) are voltage-insensitive cation channels responding to extracellular acidification. ASIC proteins have two transmembrane domains and a large extracellular domain. The molecular topology of ASICs is similar to that of the mechanosensory abnormality 4- or 10-proteins expressed in touch receptor neurons and involved in neurosensory mechanotransduction in nematodes. The ASIC proteins are involved in neurosensory mechanotransduction in mammals. The ASIC isoforms are expressed in Merkel cell–neurite complexes, periodontal Ruffini endings and specialized nerve terminals of skin and muscle spindles, so they might participate in mechanosensation. In knockout mouse models, lacking an ASIC isoform produces defects in neurosensory mechanotransduction of tissue such as skin, stomach, colon, aortic arch, venoatrial junction and cochlea. The ASICs are thus implicated in touch, pain, digestive function, baroreception, blood volume control and hearing. However, the role of ASICs in mechanotransduction is still controversial, because we lack evidence that the channels are mechanically sensitive when expressed in heterologous cells. Thus, ASIC channels alone are not sufficient to reconstruct the path of transducing molecules of mechanically activated channels. The mechanotransducers associated with ASICs need further elucidation. In this review, we discuss the expression of ASICs in sensory afferents of mechanoreceptors, findings of knockout studies, technical issues concerning studies of neurosensory mechanotransduction and possible missing links. Also we propose a molecular model and a new approach to disclose the molecular mechanism underlying the neurosensory mechanotransduction.

- Introduction
- Expression of ASICs in mechanoreceptors
  - Skin
  - Cardiovascular system
  - Visceral organs
  - Muscle
  - Joint
  - Teeth
  - Auditory and vestibular systems
- Deficit in mechanotransduction in ASIC-knockout mice
  - Electrophysiology of skin-nerve preparations
  - Behavioral studies of cutaneous mechanosensitivity
  - Visceral mechanotransduction
  - Baroreception
  - Hearing
- Probing neurosensory mechanotransduction on cell-based assays
  - Problems in knockout studies
  - The whole-cell mechano-clamp technique
  - Problems and limitation of the whole-cell mechano-clamp technique
  - Probing sensory nerve mechanotransduction via localized elastomeric matrix control
- What is missing?
  - Accessory proteins the MEC-4 channel complex
  - ECM and ECM-linker proteins
  - Tether model for the ASIC complex in neurosensory mechanotransdcution
- Conclusions

## Introduction

**Fig. 1:**
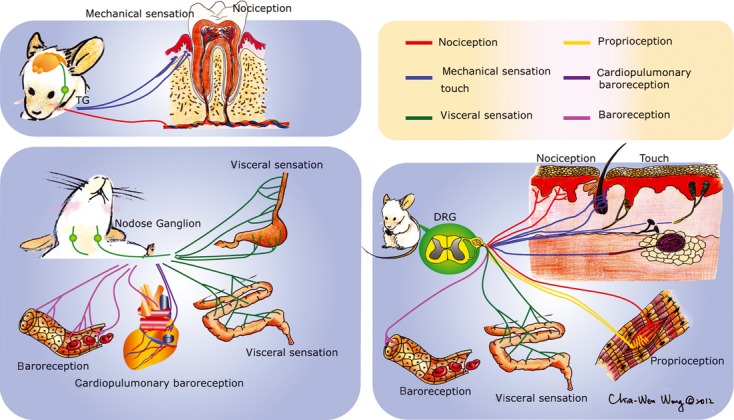
Acid-sensing ion channel (ASIC) proteins reside in nerve endings of mechanoreceptors. ASIC immunoreactivity or ASIC-like currents are found in most sensory mechanoreceptors of trigeminal, nodose and dorsal root ganglia. ASIC-expressing mechanoreceptors have diverse sensory functions ranging from nociception, touch, proprioception and baroreception, to visceral mechanosensation.

Most mammalian sensory neurons transduce mechanical forces and transmit this sensory information to the brain. Sensory neurons of the dorsal root ganglia (DRG), trigeminal ganglia and nodose ganglia detect both innocuous and noxious mechanical stimuli for sensing touch and pain or monitor stretch from muscle, vessels and visceral organs for proprioception, baroreception and interoception (Fig. 1). This neurosensory mechanotransduction is essential for regulating many physiological processes, including neurogenic inflammation, blood pressure, blood volume homeostasis, bladder voiding, stretch-evoked responses of visceral organs and proprioception. However, the mechanosensitive ion channels that allow sensory neurons to transduce mechanical stimuli are still not obvious in mammals. Studies of the nematode *Caenorhabditis elegans* support that mechanosensory abnormality 4- or 10- (MEC-4/MEC-10) proteins are sensory mechanotransduction channels [1, 2].

Mechanosensory abnormality 4- or 10- are members of a large protein family, degenerin (DEG)/epithelium sodium channel (ENaC)/acid-sensing ion channels (ASICs). The ASIC proteins are cation channels and are highly expressed in sensory neurons and thus are promising candidates of neurosensory mechanotransduction in mammals [3, 4]. Mice with null mutation of ASIC1a, ASIC1, ASIC2, or ASIC3 show abnormal neurosensory mechanotransduction [5–8]. However, the role of ASIC proteins in mammalian neurosensory mechanotransduction is still illusive, because the phenotypes found in knockout studies were subtle or controversial [4, 9].

## Expression of ASICs in mechanoreceptors

The ASICs represent a group of proton-gated ion channels of the DEG/ENaC/ASIC superfamily, which are two-transmembrane proteins assembling as a trimeric sodium channel that is amiloride sensitive and voltage independent [3]. Mammals have at least seven ASIC isoforms—ASIC1a, ASIC1b, ASIC2a, ASIC2b, ASIC3, ASIC4 and ASIC5—encoded by five genes (Accn1, Accn2, Accn3, Accn4, Accn5). The ASIC1a and ASIC1b are protein products of alternative transcription from Accn2, and ASIC2a and ASIC2b are products of alternative transcription from Accn1. The ASICs are largely expressed in primary sensory neurons and the afferents of mechanoreceptors, which indicates their roles in neurosensory mechanotransduction (Fig. 1). Here, we summarize the immunohistochemistry or electrophysiology evidence of the expression of ASIC proteins in mechanoreceptors. Of note, the interpretation of ASIC expression in peripheral afferents is cautioned, because immunohistochemistry of membrane proteins is difficult, and only some data have been validated in gene-knockout samples (Table 1).

**Table 1:** Acid-sensing ion channels (ASIC) immunoreactivity in sensory afferents of mechanoreceptors

	Expression	Species	KO	References
ASIC1	Skin			
	*Pacinian corpuscles*	H		[15]
	Cardiovascular			
	*Baroreceptors*	M	X	[18]
ASIC2	Skin			
	*Pacinian corpuscles*	H, Mo		[5, 12, 13, 15]
	*Meissner corpuscles*	H, Mo, R		
	*Lanceolate endings*	M, R	X	
	*Merkel cell complex*	R		
	*Penicillate endings*	R		
	*Nociceptors*	R		
	Bladder			
	*Suburothelium nerve complex*	R		[31]
	Cardiovascular			
	*Baroreceptors*	M	X	[18]
ASIC3	Skin			
	*Meissner corpuscles*	M	X	[6]
	*Lanceolate endings*	M	X	
	*Merkel cell complex*	M	X	
	*Nociceptors*	M	X	
	Bladder			
	*Suburothelium nerve complex*	R		[31]
	Cardiovascular			
	*Baroreceptors*	M	X	[18]
	*Volume receptors*	M	X	[19]
	Periodontal			
	*Ruffini endings*	R		[49]

KO, knockout; H, human; M, mouse; Mo, Monkey; R, rat; X, immunoreactivity was absent in knockout samples.

### Skin

The skin is innervated by a variety of sensory afferents of mechanoreceptors, where they detect a wide range of tactile qualities, such as shape, texture, vibration and pressure, as well as noxious stimulation [10]. The expression of ASIC2 and ASIC3 in cutaneous afferents and mechanoreceptors has been well studied in rodents, primates and human by immunohistochemistry [11]. The ASIC2 resides in the nerve endings of cutaneous rapid-adapting (RA) low-threshold mechanoreceptors. In glabrous skin, ASIC2 expression is restricted to axons projecting to both Meissner and Pacinian corpuscles, which indicates its role in touch sensation [12]. In hairy skin, ASIC2 immunoreactivity (both ASIC2a and ASIC2b) is found in the palisades of lanceolate nerve endings that surround the hair follicle, running longitudinally to the shaft, to detect the movement of the hair shaft [5]. However, how ASIC2a and ASIC2b contribute to the immunoreactivity of skin is not known. Controversially, one study involving ASIC2a-selective antibodies revealed distribution of the channel in both RA and slow-adapting (SA) mechanoreceptors, including Meissner, Merkel, penicillate, reticular, lanceolate and hair follicle palisades, as well as free myelinated nerve endings in rat skin [13]. Whether the discrepancy is due to antibody specificity or species is not known. Nevertheless, although this expression profile of ASIC2 would suggest a role in the transduction of touch and painful mechanical stimuli, no cutaneous pain phenotype has been reported in Asic2^−/−^ mice.

The ASIC3 is expressed in some of the free nerve endings running in the epidermal layer, which are nociceptors. In glabrous skin, ASIC3 immunoreactivity is detected in nerves of Meissner corpuscles, specialized cutaneous sensory structures located at the apex of the dermal papillae [6]. In hairy skin, ASIC3-expressing nerve endings are also found in the palisades of lanceolate nerve endings that surround the hair shaft. ASIC3 can be detected in Merkel cells or adjacent nerves.

In contrast to the expression of ASIC2 and ASIC3, that of ASIC1 in cutaneous mechanoreceptors is less addressed, although activation of ASIC1 in the mouse hind paw triggers pain behaviours [14]. A recent study revealed ASIC1 immunoreactivity in the central axon of Pacinian corpuscles in human skin [15].

### Cardiovascular system

Baroreceptors are mechanoreceptors that are sensory nerve endings in the aortic arch, venoatrial junction and carotid sinuses detecting blood pressure; they are key regulators of blood pressure and blood volume and are involved in neurohumoral control of circulation [16, 17]. There are two types of baroreceptors: high-threshold arterial and low-threshold baroreceptors (also known as cardiopulmonary or volume receptors).

The ASIC1, ASIC2 and ASIC3 immunoreactivity has been detected in aortic depressor nerve terminals in the aortic arch, the arterial baroreceptors projecting from nodose ganglia [18]. Importantly, ASIC2 immunoreactivity co-localized with ASIC1 or ASIC3 in the arterial baroreceptors, which indicates that heteromeric ASIC proteins are involved in baroreception. The ASIC2-expressing aortic baroreceptors are highly sensitive to mechanical stimulus as compared with other nodose neurons. Only ASIC3 immunoreactivity has been examined in low-threshold baroreceptors. The nerve terminals of volume receptors located in the venoatrial junction are immunoreactive for ASIC3. The ASIC3-expressing volume receptors are activated by mechanical stimuli from blood volume expansion [19]. However, the expression of ASIC1 and ASIC2 in volume receptors has not been determined.

### Visceral organs

Visceral organs receive dual afferent innervation, thoracolumbar and vagal/sacral afferents, from the same nerves as sympathetic and parasympathetic efferents. Despite few immunohistochemistry data showing the expression of ASIC proteins in visceral afferents, retrograde studies revealed that ASIC-bearing sensory neurons innervate different visceral organs, including lung, oesophagus, stomach, colon and bladder [20–23]. Spinal and vagal afferent fibres are differentially responsible for visceral pain caused by noxious distension of the gastrointestinal tract [24, 25]. Noxious mechanical stimulation on vagal afferent fibres innervating the upper abdomen causes pain and discomfort, whereas noxious distension of the colon is signalled to the brain *via* the spinal cord [26, 27].

Retrograde tracing of gastric sensory neurons reveals that all gastric DRG neurons and 55% of gastric nodose neurons express ASIC currents [27]. From the effects of Zn^2+^ and TPEN, ASIC1a and ASIC2a might contribute to ASIC currents in gastric sensory neurons. However, another study found ASIC3 immunoreactivity in 82% and 75% of stomach-innervated neurons in DRG and nodose ganglia respectively [20]. Thus, different gastric sensory neurons might express multiple ASIC isoforms, but the detailed ASIC composition has yet to be defined.

The expression of ASIC1, ASIC2 and ASIC3 in colonic sensory neurons has been determined in retrograde tracing studies with *in situ* hybridization [23]. ASIC1, ASIC2 and ASIC3 transcripts are expressed in 30%, 47% and 73%, respectively, of mouse colonic sensory neurons within thoracolumbar DRG. However, immunohistochemistry revealed ASIC3 proteins in 43% of thoracolumbar and 42% of lumbosacral labelled colonic sensory neurons in rats [28]. Surprisingly, electrophysiological study revealed only 4% of lumbosacral colonic sensory neurons expressing ASIC-like currents in mice [29].

The urinary bladder is a temporary storage organ exposed to varying volumes of urine. Sensory neurons of DRG projecting to the bladder sense the mechanical stimulation caused by bladder distension and trigger micturition [30]. Both ASIC2a and ASIC3 immunoreactivity was found in suburothelium nerve fibres, and the expression was upregulated in a model of cystitis triggered by cyclophamide [31]. Thus, both ASIC2a and ASIC3 might contribute to the neurosensory mechanotransduction involved in the micturition reflex in the normal and inflamed bladder. Future studies with ASIC-knockout mice might advance our understanding of the molecular mechanism that controls micturition in the normal bladder and with interstitial cystitis.

### Muscle

Mechanoreceptors of muscle afferents are responsible for the sensation of pain and proprioception [32–35]. Most sensory neurons projecting to muscle nociceptors and proprioceptiors show ASIC-like currents [36, 37].

More than half of muscle nociceptors are immunoreactive for ASIC3 and express ASIC3-like currents [37]. Most muscle afferent neurons that express ASIC3 (83%) are also immunoreactive for calcitonin gene-related peptide, a vasodilatory peptide, and the nerve endings project to blood vessels. The ASIC3-expressing muscle nociceptors are responsible for chronic mechanical hyperalgesia that develops after inflammation or repeated intramuscular acid injection [38–40]. However, data supporting ASIC3 as a mechanotransducer in muscle nociceptors are not yet available. As well, the expression of ASIC1 and ASIC2 in muscle nociceptors is not known.

Proprioceptors are stretch-sensitive muscle afferents that project to muscle spindles, where they provide information about the muscle length and velocity of contraction, thus contributing to an individual's ability to discern joint movement and joint position. The ASIC2 immunoreactivity was found in nerve profiles of muscle spindles [34]. Another study showed ASIC3 immunoreactivity in proprioceptors in the mesencephalic nucleus of the fifth nerve [41]. The non-selective ASIC blockers, amiloride and three analogues, at 1 mM, could largely inhibit the stretch-evoked afferent discharge in rat muscle spindles, so ASIC2 and ASIC3 may be the major contributors to proprioception [34].

### Joint

Synovial joints are sensory organs providing continuous feedback about position sensing and degree of limb movement. The transduction mechanisms require afferent nerve fibres of mechanoreceptors that convert mechanical forces acting on the joint to an electrochemical signal that transmits to the central nervous system. ASIC proteins might be one of the candidate mechanotransducers in joint afferents because amiloride (at 0.1 and 1 mM) could inhibit the joint nerve firing in response to mechanical rotation [42]. Only ASIC3 expression has been examined in joint afferents. Retrograde tracing studies revealed that 18% to 31% of knee-joint afferent DRG neurons are immunoreactive for ASIC3 [43, 44]. However, ASIC3 immunoreactivity is barely observed in joint afferents innervating the synovium unless under joint inflammation conditions [45].

### Teeth

Mechanotransduction of pulpal afferents is associated with pain in a sensitive tooth. From the hydrodynamic theory, tooth sensitivity is a result of exposed dentin with patent dentinal tubules. The perception of pain occurs when a mechanical stimulus (fluid movement) activates specific sensory nerve endings at the base of the tubule and superficial pulp tissues of a sensitive tooth [46]. One third of trigeminal ganglion neurons that project towards the tooth pulp are immunoreactive for ASIC3 [47]. Single-cell RT-PCR study revealed that ASIC3 transcripts are expressed in 67% of pulpal afferent neurons [48]. These findings suggest a role for ASIC3 in mechanotransduction in tooth sensitivity. However, the expression of other ASIC isoforms was not determined. As well, ASIC3 immunoreactivity was abundantly expressed in periodontal Ruffini endings of mouse incisors, in which a primary mechanoreceptor in the periodontal ligament connects the tooth and the alveolar bone [49].

### Auditory and vestibular systems

Hearing is a special type of neurosensory mechanotransduction. Within cochlea, auditory sensory cells (or hair cells) are located in the organ of Corti to detect sound waves and transduce the mechanical signal to receptor potential. The cochlea is innervated by sensory afferents whose cell bodies are located in the spiral ganglion. ASIC2 immunoreactivity is present in most of the spiral ganglion neurons but absent in the organ of Corti in adult cochlea. Consistently, ASIC2 contributes a major component to acid-evoked excitatory responses in spiral ganglion neurons [50]. The ASIC3 transcripts were also detected in mouse cochlea [51]. *In situ* hybridization localized ASIC3 expression to cells of the spiral ganglion and the organ of Corti [52]. Furthermore, ASIC3 immunoreactivity was found in cells of spiral ganglion, sensory nerves innerving the organ of Corti, and cells in the organ of Corti, which indicates a role of ASIC3 in mechanotransduction of the mammalian cochlea. However, the expression of ASIC1 in cochlea is not known.

The vestibular system contributes to balance in mammals and to the sense of spatial orientation *via* the semicircular canals that transduce rotation movement and otolithic organs that transduce linear accelerations. Both vestibular structures are innervated by sensory neurons of vestibular ganglia. However, the molecules that contribute to the mechanotransduction in the vestibular system are not known. Mercado and colleagues showed that vestibular afferent neurons express ASIC-like currents that are sensitive to acetylsalicylic acid, which suggests an ASIC3-containing channel [53]. In contrast, hair cells of semicircular canals did not express ASIC-like currents. Furthermore, neurons of vestibular ganglia are immunoreactive for ASIC1a, ASIC2a, ASIC2b, ASIC3 and ASIC4. The vestibular ASIC proteins might contribute to establishing the afferent-resting discharge [54].

## Deficit in mechanotransduction in ASIC-knockout mice

Gene knockout in mice is an excellent genetic model to examine the effect of an ASIC-null mutation on neurosensory mechanotransduction (Tables 2 & 3). Mice with null mutation of ASIC1a, ASIC1, ASIC2 and ASIC3 are available [5, 6, 55–57].

**Table 2:** Effect of ASIC-null mutation on neurosensory mechanotransduction in primary afferent sensory neurons

Null mutants	Cutaneous afferents	Gastroesophageal afferents	Colonic afferents	DRG	Nodose Ganglion	Reference
ASIC1a	RA =	Tension ↑	Serosal ↑			[7]
	AM =	Mucosal ↑	Mesenteric ↑			
	c-fibres =					
	SA =					
	D-hair =					
ASIC2	RA ↓ =	Tension ↓	Serosal ↑	RA =		[5, 8, 59]
	AM =	Mucosal ↑	Mesenteric =	IA =		
	c-fibres =			SA =		
	SA ↓					
	D-hair =					
ASIC3	RA ↑	Tension ↓	Serosal ↓	RA =	Gastric distension ↓	[6, 8, 64]
	AM ↓	Mucosal =	Mesenteric ↓	IA =		
	c-fibres =			SA =		
	SA =					
	D-hair =					
ASIC2				RA =		[9]
ASIC3				IA =		
				SA =		
ASIC1a	RA =					[60]
ASIC2	AM ↑					
ASIC3	c-fibres =					
	SA =					
	D-hair =					

AM, A-fibre mechanoreceptor; RA, rapid-adapting mechanoreceptor; SA, slow-adapting mechanoreceptor; IA, intermediate-adapting mechanoreceptor; DRG, dorsal root ganglion; ↑, =, ↓, responses to mechanical stimulus were increased, no change, or decrease respectively.

**Table 3:** Implication of ASICs involved in neurosensory mechanotransduction affecting physiological process and behaviours

Manipulation	Effects	Evidence	References
Asic1a^−/−^	Gastric emptying ↓	Physiology	[8]
Asic2^−/−^	Baroreflex ↓	Physiology	[18]
	Faecal pellet output ↓	Physiology	
	Noise-induced temporary hearing threshold shift ↓	Physiology	[50]
Asic3^−/−^	Sensitivity to tail pressure ↑	Behaviour	[55]
	Sensitivity to dynamic mechanical stimulation ↑	Behaviour	[61]
	Acid-induced mechanical hyperalgesia ↓	Behaviour	[38]
	Response to blood volume expansion ↓	Physiology	[19]
	Pressure-induced vasodilation ↓	Physiology	[62]
	Acoustic brainstem response ↓	Physiology	[52, 66]
Amiloride	Muscle spindle discharge ↓	Physiology	[34]
	Knee joint mechanosensitivity ↓	Physiology	[42]

### Electrophysiology of skin–nerve preparations

Single fibre recording on an *in vitro* skin–nerve preparation provides a powerful way to characterize changes in sensory modality in knockout mice (Table 2). This approach can define the phenotypes of cutaneous mechanoreceptors as RA, SA, A-fibre, c-fibre, or D-hair mechanoreceptors by the conducting-velocity and firing profiles [58]. The Asic1a^−/−^ mice showed no change in cutaneous mechanoreceptor function [7]. However, Asic2-null mutation significantly decreased the sensitivity of RA and SA mechanoreceptors [5], but another study showed no change in cutaneous mechanosensation of RA fibres in Asic2^−/−^ mice [59].

The effect of Asic3-null mutation is mixed. The Asic3^−/−^ mice have shown decreased sensitivity of A-fibre mechanoreceptors, but increased sensitivity of RA mechanoreceptors [6]. Surprisingly, simultaneous disruption of mouse Asic1a, Asic2 and Asic3 genes significantly enhanced the mechanosensitivity of A-fibre mechanoreceptors, but had no effect on other cutaneous fibres of mechanoreceptors [60]. Why single or triple ASIC gene knockout has enhanced (and discrepant) effects on cutaneous mechanosensitivity, if the channels serve as the mechanosensitive channels, is not known.

### Behavioural studies of cutaneous mechanosensitivity

Like electrophysiological studies, single ASIC gene knockout has had mixed effects on cutaneous mechanosensitivity in behaviours (Table 3). All Asic1^−/−^, Asic2^−/−^ and Asic3^−/−^ mice responded normally to punctate mechanical stimulation of the hind paw in the von Frey filament test [6, 7, 55, 57, 61]. However, Asic3^−/−^ mice were sensitive in sensing higher mechanical pressure from the tail or dynamic mechanical stimulation when a 0.74-g von Frey hair was gently stroked across the midplantar surface of the hind paw [55, 61]. The Asic2^−/−^ mice showed a normal response to dynamic mechanical stimulation. Recently, ASIC3 was found to be a neural mechanosensor of skin for pressure-induced vasodilation that protects against pressure ulcers [62]. In wild-type mice, local application of low pressure to the skin triggered vasodilation, a physiological adjustment to delay the decrease in cutaneous blood flow; the Asic3^−/−^ mice failed to respond to the local pressure and the pressure-induced vasodilation.

### Visceral mechanotransduction

Neurosensory mechanotransduction in the gastrointestinal system is important for controlling gastric coordination and emptying, colonic motility and sensing pain. Stimulation of gastric and intestinal mechanoreceptors often produces disturbed gastric coordination and delayed emptying, and colonic motility is inhibited by the autonomic reflex. Mechanical hypersensitivity of colon afferents in part underlies the chronic pain of patients with irritable bowel syndrome. Studying the molecular basis of visceral mechanotransduction is important for diseases associated with the gastrointestinal system but is challenging. Thus, ASIC-knockout mice provide good platforms to probe the molecular basis that controls the visceral mechanosensation. Like skin–nerve preparations, single-fibre recordings on an *in vitro* vagus-gastroesophageal preparation or an *in vitro* colon preparation have revealed mechanosensory deficits in mice with knockout of different ASIC isoforms. However, studies of other visceral organs are lacking, and the effects of null mutation of ASIC isoforms on visceral mechanosensation are mixed (Table 2).

Mice with null mutation of Asic1a show enhanced mechanosensitivity in all colonic and gastroesophageal mechanoreceptor subtypes [8]. The Asic1a^−/−^ mice showed increased response of tension receptors to applied graded circumferential tension (0.5–5 g) and response of mucosal receptors to applied mucosal stimuli (10–1000 mg). The Asic1a^−/−^ colonic mechanoreceptors showed a significant increase in the stimulus response function in response to graded stimuli (70–4000 mg), but no change in mechanical thresholds and spontaneous activity. As well, neurosensory mechanotransduction was slightly different in the upper *versus* lower gut with loss of ASIC1a [7]. As compared with the wild-type, Asic1a^−/−^ colonic mechanoreceptors showed changed adaptation in the early and late phases of the response, but adaption was unaffected in Asic1a^−/−^ gastroesophageal mechanoreceptors. Interestingly, studies of digestive function in conscious mice revealed significantly increased time that Asic1a^−/−^ mice needed to empty a solid egg meal, by 84%, which implies a role of ASIC1a in mechanosensory feedback in regulating gastric emptying. In contrast, faecal output was unaffected in Asic1a^−/−^ mice as compared with wild-type mice.

The effect of ASIC2 disruption on visceral mechanosensation is mixed [8]. The Asic2^−/−^ mice showed an increase in the stimulus response function of gastroesophageal mucosal-receptor sensitivity, but a decrease in tension-receptor sensitivity. Colonic mechanoreceptors are differentially affected by ASIC2 disruption. Asic2^−/−^ mesenteric afferents were unaffected in the stimulus response function, but serosal afferents were more sensitive to mechanical stimulation. Studies of digestive function in conscious mice showed Asic2^−/−^ mice with no change in solid gastric emptying time but, rather, changed lower bowel function, with a significant decrease in number of faecal outputs. However, Asic2 knockout had no effect on visceral mechanonociception [59]. Loss of ASIC2 did not affect pressure-stimulated calcitonin gene-related peptide release from the colon, with, after 10 min., the colon being inflated to 60 mmHg for 5 min.

In contrast, Asic3^−/−^ mice showed decreased mechanosensitivity in some visceral mechanoreceptors [8, 63]. Asic3^−/−^ mice showed no change in the stimulus response function of gastroesophageal mucosal afferents, but tension receptors were significantly reduced in sensitivity, with no alteration in the adaption rate or spontaneous activity. Consistently, in a study of organ preparation, ASIC3 deletion blunted mechanotransduction of nodose neurons to gastric distension at low-stimulation but not high-stimulation intensities [64]. In the lower gut, Asic3^−/−^ colonic mesenteric and serosal afferents showed significantly decreased stimulus response. However, studies of digestive function in conscious mice showed that loss of ASIC3 had no effect on gastric emptying and faecal output. In contrast, Asic3^−/−^ mice were blunted to the sensitization of colon afferent mechanosensation triggered by acidic inflammatory soup [63]. Consistently, Asic3^−/−^ mice were blunted to the intracolonic zymosan-induced behavioural hypersensitivity measured as the visceromotor response to colorectal distension, which suggests a mechanosensory role of ASIC3 in visceral nociception [65].

Thus, different ASIC isoforms seem to be involved in different visceral mechanosensation. The mechanosensory role of ASIC1a may be important in regulating gastric emptying; ASIC2 might be required for faecal output, and ASIC3 might contribute to visceral nociception.

### Baroreception

Lu and colleagues reveal that loss of ASIC2 impairs baroreflex in mice [18]. Conscious Asic2^−/−^ mice showed hypertension, exaggerated sympathetic and decreased parasympathetic control of the circulation, and decreased gain of the baroreflex, which suggests a role for ASIC2 in determining mechanosensitivity of arterial baroreceptors. In contrast, Asic3^−/−^ mice showed decreased blood volume expansion-induced diuresis and arterial atrial natriuretic protein secretion, which indicates a role for ASIC3 in determining mechanosensitivity of low-threshold baroreceptors [19]. Further study may explore whether or not activating ASIC3 could rescue the dysfunction of low-threshold baroreceptors that occurs in heart failure.

### Hearing

Two independent studies showed that Asic2^−/−^ mice had no significant hearing loss and the hearing threshold was similar to that of wild-type mice, so the channels might not be directly involved in mechanotransduction in the cochlea [50, 59]. However, Asic2^−/−^ mice were more resistant to noise-induced temporary threshold shifts than wild-type mice, which suggests a role for ASIC2 in noise susceptibility of mice. In contrast, Asic3^−/−^ mice showed age-dependent hearing loss, starting from 16 weeks old, to low frequency broad-band clicks in an auditory brainstem response test [52]. Furthermore, Asic3^−/−^ mice showed hearing loss to high-frequency tone stimulation (4-, 8-, and 16-kHz frequencies) as early as 8 weeks old, so ASIC3 might be directly involved in mechanotransduction of the cochlea [66].

## Probing neurosensory mechanotransduction on cell-based assays

### Problems in knockout studies

If ASIC proteins are the ion channels that directly underlie neurosensory mechanotransduction, mice with an ASIC-isoform null mutation should show reduced or hypo-function mechanotransduction. However, some ASIC1a-, ASIC2- and ASIC3-knockout mice show enhanced neurosensory mechanotransduction (Table 2 & 3). Thus, the ASIC-knockout phenotypes of behaviour and/or physiological processes may be compensatory effects or indirect effects of mechanotransduction. This question is not easy to answer because of the multitude of putative mechanically activated channels/receptors involved in neurosensory mechanotransduction. As well, molecular identification or manipulation of single nerves in sensory nerve recording in tissues is challenging [67–69].

The discrepancy in mechanotransduction is likely due to a relatively subtle effect of ASIC proteins on neurosensory mechanotransduction. Also, other mechanosensitive ion channels (e.g. transient receptor potential channels and Piezo proteins) might mask the effects of ASIC knockout at higher intensities of mechanical stimuli in *in vivo* behavioural or physiological assays or *in vitro* recordings of organ preparations. Therefore, an *in vitro* cell-based method might be necessary to reveal the ASIC-mediated mechanotransduction.

### The whole-cell mechano-clamp technique

Many techniques have been developed to probe mechanotransduction in cells [70]. The whole-cell mechano-clamp technique is the most popular way to probe mechanically activated currents on dissociated sensory neurons. The device comprises a whole-cell patch clamp recording system and a piezoelectric actuator mounted on a 3-D micromanipulator. In the mechano-clamp, the mechanical probe is a heat-polished glass pipette that is fixed on a pipette holder. The mechanical probe was used to indent the cell soma controlled by a piezo-electric crystal drive [71]. This technique also involves fusion of a glass micropipette to the surface of the cell membrane for high-resolution recording of the whole-cell channel current through the membrane. In voltage clamp mode, mechanosensitive currents are activated gradually as a function of mechanical displacement depth. With this approach, ∼50–60% of small-diameter DRG neurons show three major mechanically activated currents: RA, intermediate-adapting (IA) and SA currents, many of which are nociceptors [72, 73]. One might challenge that the mechanically activated currents from neuron soma are not physiologically relevant to the mechanotransduction on sensory nerve terminals. A study of mechanically activated currents on neurites of dissociated DRG neurons revealed all RA, IA and SA conductance found in similar populations of sensory neurons [74]. The difference is that almost all sensory neurites process currents by mechanical displacement. The study also indicated that neurons with RA conductance might be low-threshold mechanoreceptors because they were mainly identified in large sensory neurons with narrow action potential, whereas neurons with IA or SA conductance might be nociceptors because they are exclusively expressed in small sensory neurons with longer action potential. Of note, RA, IA, SA currents identified in dissociated DRG neurons and RA, IA, SA mechanoreceptors recorded in skin–nerve preparations (or vagus-gastroesophageal and colon preparations) should not be directly compared.

### Problems and limitations of the whole-cell mechano-clamp technique

Drew and colleagues used the whole-cell mechano-clamp approach to examine mechanically activated currents in two different subsets of acutely dissociated DRG neurons [9]. The wild-type and ASIC2-, ASIC3- and ASIC2-3 knockout models did not differ in expression of RA, IA and SA in both peptidergic and non-peptidergic small-medium DRG neurons [9]. Consistently, the non-selective ASIC blocker benzamil, 100 μM, had no effect on the mean amplitude of neurite mechanically activated currents but markedly increased the latency for current activation [74]. Thus, another ion channel type but not ASICs may be important for the mechanotransduction measured by whole-cell mechano-clamp.

Coste and colleagues recently used the whole-cell mechano-clamp approach with RNA interference to clone two novel mechanosensitive ion channels, Piezo1 and Piezo2, which indicates the power of the technique to understand neurosensory mechanotransduction [75]. However, the whole-cell mechano-clamp technique has some limitations in detecting subtle mechanical stimuli, especially for low-threshold mechanoreceptors, because cells are plated on coverslips coated with poly-L-lysine mixed with laminin and the mechanical stimulus is directly applied on the cell surface. From a biomechanics aspect, this approach fails to address important structural-related challenges because the DRG neurons are cultured on hard substrates (e.g. glass) and the elastic modulus is at least several hundred times higher than in their physiological surroundings. As well, direct displacement on the cell membrane would deliver a non-specific force on the cell, lose the extracellular matrix (ECM) ligand selectivity, and damage the cell. Both ECM selectivity and cytoskeletal structure play important roles in neurosensory mechanotransduction [76–80].

### Probing sensory nerve mechanotransduction *via* localized elastomeric matrix control

**Fig. 2:**
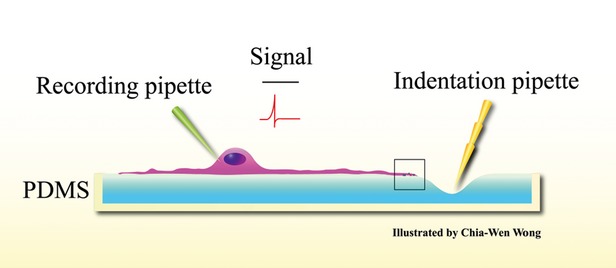
Probing sensory nerve mechanotransduction *via* localized elastomeric matrix control. Mechanical stretching imposed on a neurite *via* surface-modified elastomeric matrices. To probe the extracellular matrix (ECM)-tethered mechanotransduction on nerve terminals, neurite-bearing dorsal root ganglia neurons are cultured on an ECM-coated polydimethylsiloxane substrate that mimics a physiologically relevant elastic modulus (10–100 kPa). We can use whole-cell patch clamp recording to measure the electrical responses of neurosensory mechanotransduction on a single neurite by substrate indentation at a location adjacent to the neurite, which will deform the substrate and thus stretch the neurite without contacting it. This figure was modified from [82].

To examine neurosensory mechanotransduction *in vitro*, the choice of a cell-base technique is critical for testing the hypotheses grounded in mechanobiology [81]. To probe neurosensory mechanotransduction, we recently developed a cell-based method to culture neurite-bearing DRG neurons on an ECM-coated elastomeric substrate [82]. In such conditions, we can stretch a single neurite by substrate indentation without contacting the cell membrane. We can use whole-cell patch clamp recording to probe the neurite-stretch-induced mechanotransduction within a defined substrate, whose physical and molecular context can be modified to mimic physiologically relevant conditions (Fig. 2). This cell-based technique allows us to decode the ECM-tethered mechanosensitive ion channels. Accordingly, the neurite-stretch-induced action potential was present only in specific subsets of DRG neurons [78]. Further studies on ASIC-null mutations would be promising in determining ASIC isoforms as the ECM-tethered mechanosensitive ion channels in sensory nerves.

## What is missing?

Recently identified Piezo proteins are pore-forming subunits of mechanically activated channels in mammals [75, 83]. In flies, DmPiezo and Ppk (DEG/ENaC) function in two parallel pathways in multiple dendritic sensory neurons controlling the noxious response to harsh mechanical stimulation [84]. Expression of the fly Piezo protein in human cells induces mechanically activated currents like their mammalian counterparts. However, the expression of Ppk in heterologous cells cannot produce a stretch-activated channel current [85]. Similarly, no mammalian ASIC isoform can produce a stretch-activated channel current in heterologous expression systems. Thus, ASIC proteins are not sufficient, and some accessory proteins are missing in reconstructing the mechanotransducers in mammalian sensory mechanotransduction.

### Accessory proteins and the MEC-4 channel complex

From the molecular model of touch in nematodes, the mechanotransducers should include the amiloride-sensitive DEG/ENaC channels (MEC-4 and MEC-10), accessory proteins that enable channel activity (MEC-2 and MEC-6), cytoskeletal structure proteins (MEC-7 and MEC-12), and ECM proteins (MEC-1, MEC-5, and MEC-9) [1]. MEC-2, MEC-4 and MEC-6 co-localize in a punctate pattern along the touch receptor process to form a channel complex in *C. elegans* and are thus named the MEC-4 complex [86–88]. Null mutation in *mec-2*, *mec-4*, or *mec-6* gene eliminates the mechanoreceptor currents [2].

We speculate ASIC proteins may act together with two accessory proteins such as the MEC-4 complex for mechanotransduction in mammalian sensory neurons. Both MEC-2 and MEC-6 affect the lipid environment of the channels and thus channel activity in *C. elegans*. MEC-2 is a stomatin-like protein, which is 65% identical to stomatin in its central stomatin-domain, whereas MEC-6–like proteins in human are paraoxonases that regulate cholesterol oxidation in high-density-lipoprotein particles. When MEC-4 is co-expressed with MEC-2 or MEC-6, the amplitude of amiloride-sensitive currents are largely enhanced [86, 87].

The mammalian members of the stomatin family include stomatin; stomatin-like protein-1, -2 and -3; and podocin, all of which share 40–84% sequence similarity in the stomatin domain [89]. Mice lacking stomatin show reduced sensitivity to mechanical stimulation in a specific subset of mechanoreceptors, the D-hair receptor, in the skin [90]. Mice with null mutation of stomatin-like protein-3 show loss of mechanosensitivity in about 35% of skin mechanoreceptors and reduced tactile discrimination capability [91]. However, with overexpression, both stomatin and stomatin-like protein-3 confer reduced but not enhanced ASIC-mediated pH-gated currents [92, 93]. How the stomatin-domain proteins modulate mechanotransduction *via* ASIC proteins is not clear.

### ECM and ECM-linker proteins

In the MEC-4 complex model, ECM (MEC-5) and ECM-linker proteins (MEC-1 and MEC-9) are essential in nematode touch sensation, but whether they can directly interact with ASIC proteins in mammalian sensory nerves is not known [1].

A MEC-5 is collagen in mammal. Collagens are ECM proteins, and 29 types of collagens have been reported, including some sensory neuron-specific collagens [94]. We previously demonstrated that ECM signalling is essential for sensory nerve mechanotransduction evoked by neurite stretching on an elastomeric substrate [78]. However, limited data are available of the roles of specific collagens (or ECM proteins) in MEC-4 mechanotransduction. In rat hairy skin, integrin α2β1 signalling affects mechanotransduction in both SA and RA cutaneous mechanoreceptors, and this indicates that integrin ligands (collagens or laminin-111, -211, -511) are required in the neurosensory mechanotransduction [76]. In contrast, laminin-332 selectively suppresses the RA current in dissociated DRG neurons, which suggests that different ECM proteins might have diverse roles in neurosensory mechanotransduction [80].

The ECM-linker proteins (MEC-1 and MEC-9) with multiple endothelial growth factor domains are required for mechanotransduction in *C. elegans*, although they are less addressed in the MEC-4 model. In mammals, one of the candidates might be USH2A protein, which is a transmembrane protein with a very large extracellular domain binding to collagen IV [95]. Human genetics studies have shown that recessive pathogenic mutations in the USH2A gene impair both touch and hearing acuity [96]. The other possible candidates might be matrilin proteins. Matrilins are widely expressed in the ECM of different tissues and are involved in both collagen-dependent and -independent filament networks [97]. Donier and colleagues used yeast two-hybrid assay to identify ASIC interacting proteins and found the ECM-linker protein matrilin-2 interacting with ASIC3 [98]. Matrilin-2 contains 10 endothelial growth factor-like motifs, which are related to MEC-1 and MEC-9 [1, 99]. Interestingly, matrilin-1 and -3 play important roles in regulating chondrocyte mechanotransduction [100]. The content of matrilins is essential for optimal activation of chondrocytes by mechanical signals. Future studies should investigate the role of USH2A and matrilin-2 in ASIC-mediated neurosensory mechanotransduction.

### Tether model for the ASIC complex in neurosensory mechanotransduction

**Fig. 3:**
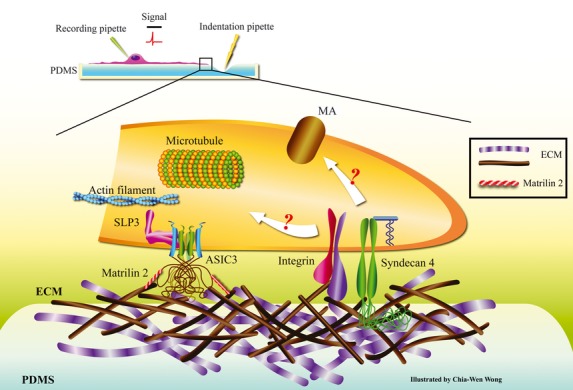
Schematic model of the ASIC complex in mediating mechanotransduction on sensory afferents**.** From the concepts of Chalfie (2009), we propose a tether model for the stretch-activated ion channel in the mammalian sensory nerve. Proteins that are needed in neurosensory mechanotransduction include the stretch-activated ion channel (e.g. ASIC3), ECM (collagens) and ECM-linker proteins (matrilin-2), intracellular linker proteins (e.g. stomatin-domain protein stomatin-like protein-3 [SLP-3], and cytoskeleton proteins (actin filament and microtubule). Integrin/syndecan-4 signalling may act in parallel to open another mechanically activated channel (MA) or modulate the ASIC complex.

From the current evidence, we propose that ASIC proteins might be the low-threshold mechanosensitive channels in specific subsets of cutaneous, muscular and visceral afferents in mammals. The ASIC-mediated mechanotransduction should act in a tether model like the MEC-4/MEC-10 complex. To activate ASIC-mediated mechanotransduction, linker proteins tethered to ECM and cytoskeletal structure would be required to transmit the mechanical signal to nerve terminals and open the ASIC channels (Fig. 3). The intracellular linker proteins should include the stomatin-like proteins, because stomatin-domain proteins can directly modulate ASIC activity in a heterologous expression system [92, 93, 101]. In addition, other linker proteins (e.g. microtubule-associated proteins and/or actin-binding proteins) might be necessary to connect ASIC proteins to the cytoskeletal structure, because the MEC-4 complex does not directly connect to the cytoskeletal structure in *C. elegans* [102]. The extracellular linker protein should contain matrilin-2 because it is an ECM-linker protein and binds to ASIC3 [98].

Alternatively, ECM-linker proteins might interact with integrin/syndecan-4 signalling and then activate downstream effector channels in transmitting mechanical signals from the microenvironment to sensory nerve terminals. Syndecan-4 is a co-receptor in adhering to a range of ECM ligands, modifying the primary integrin-mediated response, and initiating cellular mechanotransduction [103, 104]. Integrin signalling can modulate a newly identified mechanically activated channel, Piezo, but the interaction with ASIC has yet to be defined [105].

## Conclusions

ASIC1, ASIC2 and ASIC3 seem to have different roles in neurosensory mechanotransduction and are involved in many pathological processes, including neurogenic inflammation, chronic pain, hypertension, heart failure, gastroesophageal reflex disease, irritable bowel syndrome, interstitial cystitis, hearing loss, tooth sensitivity and body balance. Selective or non-selective ASIC inhibition is based on inhibiting acid-induced currents, which does not necessarily act on the ASIC complex that mediates mechanotransduction in sensory afferents [3, 4, 106, 107]. Drug screening targeting specific ASIC-mediated neurosensory mechanotransduction is a new direction for translation medicine. However, direct evidence to demonstrate ASIC proteins are the mechanically activated channels is still lacking. We propose to test the ASIC complex based on the tether model and probe the ASIC-mediated sensory nerve mechanotransduction within a defined substrate, whose ECM content and substrate elasticity are modified to mimic physiologically relevant conditions.
